# The frequency of hippocampal theta rhythm is modulated on a circadian period and is entrained by food availability

**DOI:** 10.3389/fnbeh.2015.00061

**Published:** 2015-03-11

**Authors:** Robert G. K. Munn, Susan M. Tyree, Neil McNaughton, David K. Bilkey

**Affiliations:** ^1^Department of Psychology, University of OtagoDunedin, New Zealand; ^2^Department of Neurobiology, Stanford UniversityStanford, CA, USA

**Keywords:** hippocampus, theta, food entrainable oscillator, episodic memory, circadian rhythm

## Abstract

The hippocampal formation plays a critical role in the generation of episodic memory. While the encoding of the spatial and contextual components of memory have been extensively studied, how the hippocampus encodes temporal information, especially at long time intervals, is less well understood. The activity of place cells in hippocampus has previously been shown to be modulated at a circadian time-scale, entrained by a behavioral stimulus, but not entrained by light. The experimental procedures used in the previous study of this phenomenon, however, necessarily conflated two alternative entraining stimuli, the exposure to the recording environment and the availability of food, making it impossible to distinguish between these possibilities. Here we demonstrate that the frequency of theta-band hippocampal EEG varies with a circadian period in freely moving animals and that this periodicity mirrors changes in the firing rate of hippocampal neurons. Theta activity serves, therefore, as a proxy of circadian-modulated hippocampal neuronal activity. We then demonstrate that the frequency of hippocampal theta driven by stimulation of the reticular formation also varies with a circadian period. Because this effect can be observed without having to feed the animal to encourage movement we were able to identify what stimulus entrains the circadian oscillation. We show that with reticular-activated recordings started at various times of the day the frequency of theta varies quasi-sinusoidally with a 25 h period and phase-aligned when referenced to the animal’s regular feeding time, but not the recording start time. Furthermore, we show that theta frequency consistently varied with a circadian period when the data obtained from repeated recordings started at various times of the day were referenced to the start of food availability in the recording chamber. This pattern did not occur when data were referenced to the start of the recording session or to the actual time of day when this was not also related to feeding time. This double dissociation demonstrates that hippocampal theta is modulated with a circadian timescale, and that this modulation is strongly entrained by food. One interpretation of this finding is that the hippocampus is responsive to a food entrainable oscillator (FEO) that might modulate foraging behavior over circadian periods.

## Introduction

The function of the hippocampus has previously been linked to processes underlying episodic-like (what, where, when) memory (Aggleton and Brown, 1999; Eichenbaum and Fortin, 2003; Kesner and Hunsaker, 2010). While the evidence for hippocampal representation of “where” (O’Keefe and Nadel, 1978) and “what” (Bostock et al., 1991; Hok et al., 2007; Barry and Muller, 2011) is considerable, most data pointing to a role in encoding “when” an event occurred has been obtained across short delay intervals (Meck et al., 1984; Fortin et al., 2002; MacDonald et al., 2011; Eichenbaum, 2014). It is, however, of considerable interest to understand how the hippocampal formation might represent time at longer, behaviorally relevant, timescales. Time of day is central to the foraging strategies of animals as diverse as small rodents (Jackson, 2001), fish (Reebs, 1996), and birds (Biebach et al., 1991; Meyers-Manor et al., 2014). Given this remarkable conservation of function, it is reasonable to suppose the episodic memory system has evolved with the capacity to resolve episodes with day-long resolution. Laboratory animals display robust food-anticipatory increases in activity when they are fed at regular intervals (Edmonds and Adler, 1977; Escobar et al., 2009), suggesting the successful coding of information on very long timescales. Our recent work has shown that the firing rate of single neurons in the hippocampus is modulated on a circadian timescale (Munn and Bilkey, 2012), providing a potential mechanism by which the hippocampus could represent longer durations. Unlike most other circadian rhythms, this modulation is not entrained to light. It was, therefore, proposed that the availability of food might be an entraining stimulus (Munn and Bilkey, 2012), in line with myriad number of findings suggesting the presence of a food-entrainable circadian oscillator outside of the traditional light-entrained oscillator driven from the suprachiasmatic nucleus (Phillips et al., 1993; Stephan, 2002; Landry et al., 2006; Mistlberger, 2006; Storch and Weitz, 2009; Verwey and Amir, 2009; Mendoza et al., 2010; Silver and Balsam, 2010). Findings that the hippocampus is involved in the regulation of energy state (Davidson and Jarrard, 1993); that restricted feeding produces a localized phase shift in the expression of the clock genes mPer1 and mPer2 in the hippocampus (Wakamatsu et al., 2001), and that the hippocampus is necessary for animals to solve a task where energy state is the discriminative cue (Davidson et al., 2010) are consistent with the hypothesis that the hippocampus has a role in encoding circadian-scale information about food. This would locate the hippocampus in a distributed circuit as part of the putative food-entrainable oscillator (Carneiro and Araujo, 2009).

As previous data links activity in the hippocampus to detection of contextual and/or spatial novelty (Mumby et al., 2002; Wills et al., 2005; Hoge and Kesner, 2007), an alternative entraining stimulus for the circadian modulation of hippocampal single unit activity is the initial exposure to the recording environment. Differentiating between novelty or food as an entraining stimulus is problematic when recording either hippocampal theta or from single CA1 place cells because animals have to be encouraged to move through the recording environment to ensure animals traverse the place field of the cell of interest (in the case of single cells) and, since theta is strongly associated with voluntary movement, to ensure the reliable generation of theta in the local EEG (O’Keefe and Dostrovsky, 1971; O’Keefe and Conway, 1978). Animals are typically encouraged to explore the environment via the random scattering of food throughout the behavioral arena. In the present study we disambiguated food from environment entry by using hippocampal 4–12 Hz EEG (theta) activity as a proxy for the underlying neural activity (Buzsàki and Eidelberg, 1983; Fox et al., 1986; O’Keefe and Recce, 1993; Skaggs et al., 1996; Hasselmo, 2005; Foster and Wilson, 2007). Once we had demonstrated that freely-moving theta activity displayed a circadian modulation we could then use stimulation of the reticular area to produce a form of theta rhythm in the hippocampus that can be recorded, and indeed must be recorded, during immobility (McNaughton and Sedgwick, 1978; McNaughton et al., 1986; Vinogradova et al., 1998). We demonstrated that reticular-activated theta (RAT) is also modulated over a circadian period. Using this paradigm, we were then able to systematically disambiguate time of day, environment entry, and food availability as stimuli that entrained this modulation. We show for the first time that circadian modulation of this hippocampal activity is entrained by food.

## Materials and Methods

The procedures described in these experiments were approved by the Otago committee on ethics in the care and use of laboratory animals, and complied with the University of Otago code of ethical conduct.

### Experiment One

#### Subjects and Surgery

The subjects in experiment one were eight male Sprague-Dawley rats that weighed between 400–600 g and were approximately 5 months old at the time of surgery. These animals were implanted with sixteen-channel driveable microelectrode arrays. These arrays were an adaption of the drive style described by Bilkey and Muir (1999) and the electrodes were targeted at the dorsal CA1 cell layer as described in Munn and Bilkey (2012). The EEG data from three of these animals were recorded coincident with the single unit recordings reported in Munn and Bilkey (2012), while the remaining five animals were new to study. Animals were allowed twelve days to recover following surgery before the beginning of recording. These animals were housed in the same animal room as described in Munn and Bilkey (2012). Briefly, animals were housed in single cages (33 cm × 19 cm × 26.5 cm) at a consistent, thermostatically controlled 20–22 degrees Celsius. Animals were held under strict 12:12 light:dark conditions with lights on at 0600 and lights off at 1800 h and were not exposed to natural light. Animals had *ad libitum* access to water, and were fed a set amount of food daily at 1700 h. The amount of food each animal was given was adjusted on a daily basis to maintain the bodyweight of each animal as close as possible to 85% of their weight when free-feeding.

#### Histology

Animals were deeply anesthetized with halothane, and were perfused transcardially with 0.9% saline and then with 10% formalin. The brains were removed and stored for several weeks in 10% formalin. Brains were rapidly frozen and then sectioned coronally into 40 micron slices by cryostat. Slices were mounted and stained using thionin. Electrode placement was confirmed visually under microscopy.

#### Apparatus

The recording apparatus and data acquisition were as described in Munn and Bilkey (2012). Briefly, animals were connected to a head stage cable attached to a commutator, which was in turn connected to an Axona DACQUSB system that acquired and digitally amplified the recordings. A 48 kHz sampling rate was used for single unit recording and EEG was sampled at 4800 Hz. Animals were routinely screened daily for single units (40 min per day) in the recording apparatus. Depending on the animal, they had between 5 and 10 40-min sessions in the recording apparatus before the long recording sessions from which the experimental data are derived. During initial pre-screening, all channels were checked for signals and the clearest, most noise free channel was selected for EEG recording. This channel was then simultaneously high and low pass (0–500 Hz) filtered during the long exposure recordings. The low pass filter also included a notch filter at 50 Hz to remove AC noise. The recording chamber was a black-painted wooden box measuring 61.5 × 61.5 × 60 cm. A small (7 × 5.5 cm) white card was attached half way along the Northern wall of the box at the top. The recording room was kept in constant dim light throughout the whole procedure and attention was paid to eliminating uncontrolled time-of-day cues. Each hourly recording was triggered by a custom-written program which kept track of time and sent the necessary key presses to the DACQUSB software to begin each recording on the hour. The Axona system controlled a mechanical feeder affixed to the wall 30 cm above the recording chamber. A custom-written script activated the feeder for 1.5 s every 30 s during each recording session.

#### Procedure

Animals were placed into the recording environment and an initial 20 min long recording was started. During this period food pellets were scattered randomly throughout the environment from an automated feeder at a frequency of 1–2 pellets every 30 s for the duration of the recording in order to encourage movement. The feeder was modified from a Campden Instruments Ltd. operant chamber pellet dispenser, and the pellets themselves were standard 40 mg pellets (Reliance Stock Foods Ltd.) Animals remained in the recording environment, where they were free to behave, for between 25 to 30 h and the 20 min recording procedure was repeated every hour for the duration of this period. The recordings all started at different times of day (1100, 1700, 1630, 1730, 1130, 1500, 1530, 1300, 1600, 1200, 13:30, and 1500 h). Data recorded during each session were saved for off-line analysis.

#### Data Collection and Analysis

The 20 min long EEG trace from each animal’s hourly recording session was analyzed using a custom-written MATLAB script. A fast Fourier transform was performed on the data in order to generate a power spectrum for the whole 20 m recording. The frequency of peak power in the theta-band (4–12 Hz) range was thereby determined for each recording. The movement of the animals was determined by tracking their head location using two LED lights attached to the headstage. The Axona system tracked the *x* and *y* positions of these lights at a 50 Hz sampling frequency. Speed was calculated at 100 ms intervals and averaged with a moving average across 200 ms blocks throughout the recordings. The amount of time the animal spent moving at a given speed was binned *post hoc* into 2 cm/s wide velocity bins. If animals spent more than 20% of the recording session in the 0–2 cm/s recording bin, that hour’s recording was discarded. Data was also discarded if an animal covered less than 60% of the environment. This ensured that animals were moving throughout most of each 20 m recording, and were therefore likely not asleep (Munn and Bilkey, 2012). The theta frequency for each hour was averaged over recording sessions, producing a mean frequency at maximum power for all recording sessions. Circular statistics were used in an initial analysis to determine whether there was a statistically significant level of modulation in the data across a circadian period of 25 h. This particular period was chosen because previous evidence suggests that the circadian rhythm tends to run at this period in animals that are deprived of time-of-day cues (Honma and Hiroshige, 1978). The use of circular statistics across a relatively small number of cycles (in this case one) requires equal sampling of all phase angles and so data were truncated at 25 h and missing data were replaced by group means. Frequency values were normalized to between zero and one and entered into Moore’s Modified Rayleigh Test (Oriana, Kovach Computing Services). Following this procedure, a series of sine waves with 25 h period and peak offset varying between −8 to +8 h prior to recording start was fitted to the mean data to determine phase offset. These analyses supported our decision to use the reference sine wave that generated the best fit in our previous single unit data; a 25 h period sine wave with a positive going peak 2 h into recording (Munn and Bilkey, 2012) in subsequent analyses where the mean frequency at maximum power in each hour of recording was correlated against this reference as a constrained measure of circadian periodicity. A partial correlation analysis was performed in SPSS to determine the contribution of movement speed to the correlation between the mean frequency at maximum power and the sine wave.

#### Data Shuffling

Data were shuffled by generating a pseudo-random number series from 1-n, where *n* was the number of hours in the dataset, without replacement. These random number series were used as a key to shuffle the data time-series obtained from the long-duration recordings. This procedure was repeated 1000 times in order to produce 1000 shuffled versions of the dataset which were then correlated against the reference sine wave in the same way as the actual data.

### Experiments Two and Three

#### Subjects and Surgery

The subjects in these experiments were six male Sprague-Dawley rats that weighed between 300–450 g and were approximately 3 months old at the time of surgery. Animals were chronically implanted with a bipolar recording electrode located at the border between CA1 and the dorsomedial subiculum and with a bipolar stimulating electrode located in the region of nucleus reticularis pontis oralis, as per our previous studies (McNaughton and Sedgwick, 1978; McNaughton et al., 1986; Munn and McNaughton, 2008) using coordinates derived from Paxinos and Watson (1998). These coordinates were −5 mm posterior to bregma, 2.5 mm lateral from the midline and 5 mm deep (Recording); −6 mm posterior, 1.8 mm lateral and 7.5 mm deep (Stimulating). General surgical techniques including anesthesia and supportive drug usage were as described in Munn and Bilkey (2012). Animals were housed individually in an otherwise blacked out home room that was maintained on a 12:12 light:dark schedule with lights on at 0600 and off at 1800. On initial admission into the home room, animals had access to food and water *ad libitum*. From the time immediately prior to surgery, however, animals were given a pre-determined quantity of food at precisely 1800 h every day. The amount of food was adjusted to maintain animals at approximately 85% of their free feeding weight at all times. Histological procedures were as described in experiment one.

#### Apparatus

Stimulation was produced by a programmable stimulator which delivered a 1000 ms burst of monophasic 0.1 ms pulses at 100 Hz. This stimulator was controlled by an IBM PC compatible computer running custom software. EEG was amplified by a Grass P511 amplifier before being passed to a Cambridge instruments 1401 DACQ which sampled the EEG at 128 Hz. This device interfaced with the PC and data was acquired, displayed in real-time and saved for later analysis via a custom script written in Spike 2 (Cambridge Instruments). The recording room in which the recording sessions took place was dimly illuminated at all times by a hooded 60 W light bulb angled toward the ceiling.

#### Procedure

##### Stimulation of Reticular Activated Theta (RAT)

Prior to the start of experimentation, animals were tested for the quality of RAT by connecting them to the recording apparatus and stimulating the reticular formation with trains of ascending current intensity while they were motionless. The stimulation intensity that elicited theta differed between animals due to differences in the proximity of the stimulating electrode to the reticular formation. Animals that had clear RAT were then tested to determine the maximum and minimum stimulus intensities. The maximum intensity was that which produced significant head turning or clear 9 Hz RAT. The minimum intensity was that which reliably produced clear RAT of any frequency. Six evenly spaced stimulation intensities were then selected across this range, and these values were used during the subsequent experimental sessions. During the experiment proper, the animals remained in the recording environment for a period of 49 h (experiment two) or 59 h (experiment three). Experiment three was of longer duration than experiment two to allow for the recording of data for more than two full solar days. A recording session was initiated immediately upon the animal’s entry into the recording environment, and thereafter for every hour from first entry. Each recording session consisted of four repetitions (two ascending and two descending) of the set of stimulation intensities determined earlier that produced clear RAT with no head turning or movement artifact. All stimulation bursts were delivered manually, when the experimenter had determined the animal was motionless and awake. The experimenter manually determined that the animal was awake if it was awake with eyes open and alert before each stimulation. If the animal appeared asleep, the animal was gently prodded by the experimenter to wake it up. Each individual burst of stimulation was delivered as soon as was practical after the last (it was occasionally necessary to wait for the animal to become quiescent after stimulation). Each recording session ran for approximately 10 min total; typically not less than eight and not more than fifteen minutes. Animals had access to water *ad libitum* throughout all recording trials. Between stimulation epochs, animals were free to sleep, groom, or behave however they wished.

### Experiment Two

Three naive animals participated in experiment two. Each of these animals was run through the procedure twice, providing a total of six recording sessions. Each one of these sessions consisted of 49 stimulation runs, each occurring an hour apart. The animals were in the apparatus for the whole 49 h period but started their recording sessions at different times of the day (1700, 0500, 2200, 1000, 1200 and 0000 h). Runs were 12 h-matched within animals; e.g., if an animal’s first run was at 1700 its second run was at 0500), and animals were consistently fed at exactly 1800 h irrespective of the starting time of their runs.

### Experiment Three

Three naive animals participated in experiment three, and were each run through the procedure twice, as in experiment two, generating six 59-h data-series. This experiment was identical to experiment two except that animals were fed once at the beginning of their experimental sessions irrespective of normal feeding time, and thereafter at 24 h intervals. Experimental sessions for these animals lasted for 59 h and the animals spent this whole period in the recording apparatus.

#### Data Collection and Analysis

The 20 stimulation-elicited EEG waveforms captured in each hour’s session were subjected to fast Fourier transform using a window width of 128 samples in Spike 2 using a custom written script. This produced a power spectrum in the 1–12 Hz frequency band that was divided into 1 Hz bins. The power values in each 1 Hz bin were then collapsed across stimulation intensity, producing a single power value in each of the frequency bins for each hour of recording. The power in each of the 7, 8, and 9 Hz bands was then further collapsed in order to produce the mean power value for each recording across this band.

The mean frequency of RAT for each stimulation strength in each hourly stimulation session was determined by multiplying the power in each of the 1 Hz frequency bands by the frequency of that band, adding these values and then dividing this value by the sum of the power values in each frequency bin. The overall average frequency in each recording session was therefore derived from the average frequency of all five stimulation intensities in each session. For each of the six runs, this frequency value was normalized by dividing the frequency in each hour by the maximum frequency observed over the entire recording run. This normalization was performed to enable meaningful analysis over all six runs in each experiment, since the stimulation intensities necessarily varied from animal to animal. Data shuffling was conducted as described for experiment one.

A Power spectral density (PSD) estimate of the frequency data time series was determined using the pwelch function in MATLAB applied with a sampling frequency of 25 h. The peak frequency was extracted from the PSD within a 12.5–50 h window. One of the most common methods of determining rhythmicity in circadian datasets is cosinor analysis; a least-squares regression model that fits cosine functions of a given wavelength and variable offset to a data series, provides the characteristics of the wave of best fit, and determines the goodness of fit. This procedure was carried out in MATLAB (v R2013b) using a freely available cosinor analysis script (Nelson et al., 1979).

## Results

### Circular Statistics and Correlations with Reference Sine

#### Experiment One—Free-Running Theta Frequency at Maximum Power

In order to assess periodicity in the data set at the predicted 25 free-running period we entered individual trial data, truncated at 25 h, into a Moore’s modified Rayleigh test, where frequency was represented as vector length at each phase angle. The results indicated significant periodicity (*R* = 1.1, *p* < 0.05) with a mean phase angle of 341 degrees, placing the acrophase close to the recording start. To further characterize acrophase we fitted a series of sines with 25 h periods and variable phase delay to the mean data. This analysis showed that significant fits were obtained when the reference peak occurred from between 3 h before, to 4 h after, recording start.

**Figure 1:**
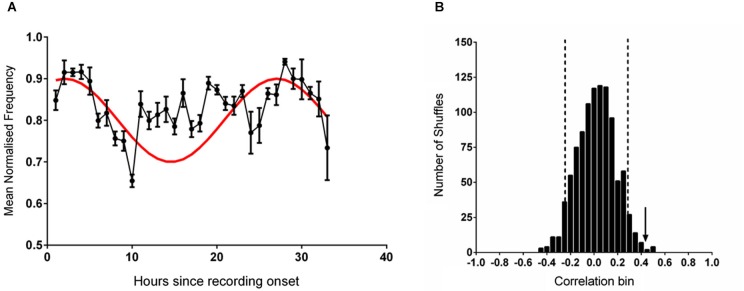
**(A)** The mean (±sem) normalized frequency of spontaneously generated hippocampal theta rhythm during each hour of recording (black line). The reference sine wave used for comparison is shown in red. **(B)** The frequency distribution produced by correlating 1000 shuffled versions of the data shown in **(A)** against the reference sine. The vertical dashed black lines demark two standard deviations from the mean of the distribution. The vertical black arrow indicates the magnitude of the correlation of the unshuffled data shown in **(A)** against the reference sine.

It has previously been established that the firing rate of CA1 place cells is associated with features of hippocampal theta (Buzsàki and Eidelberg, 1983; Fox et al., 1986; O’Keefe and Recce, 1993; Skaggs et al., 1996; Hasselmo, 2005; Foster and Wilson, 2007). To test whether this relationship between theta and cell activity existed in the present dataset the mean theta frequency in each hour of recording was compared with the mean normalized firing rate of place cells recorded from the same animals and previously reported in Munn and Bilkey (2012); there was a very strong correlation between these two datasets (*r* = 0.907, *p* < 0.001). In our previous study (Munn and Bilkey, 2012) we had demonstrated that the mean firing rate of hippocampal place cells, was modulated at circadian periods and that this data was best fitted to a sinusoidal wave with a period of 25 h and a positive peak 2 h after recording onset. Given the relationship between firing rate and theta frequency observed in our data and the results of the initial analysis that indicated an acrophase in the theta data at or near to the recording start we set up the *a priori* hypothesis that guided all subsequent data analyses; that the theta frequency dataset would be well-fitted by a sinusoid with a 25 h period and peak 2 h into the recording, when the individual data were referenced to the proper entraining stimulus. This procedure markedly reduced the degrees of freedom in our analysis. When a comparison was made between this reference sine and normalized theta frequency as determined at the point of maximum power within the theta band a significant correlation was apparent (*r* = 0.414, *p* = 0.02; Figure 1A). This association decreased slightly but remained significant when the contribution of movement speed (determined from the average speed in each recording hour) was partialled out (*r* = 0.404, *p* = 0.026). Mean running speed itself was not significantly correlated with mean theta frequency (*r* = 0.088, *p* = 0.637, n.s) or with the sine reference (*r* = 0.223, *p* = 0.228, n.s).

When the individual datasets were aligned to solar time of day and averaged no rhythmicity was evident and the fit to the reference 25 h sine was poor (*r* = −0.009, *p* = 0.961). When the data were shuffled 1000 times and individually correlated against the sine reference (Figure 1B) a normally distributed set of correlation values was generated (Shapiro-Wilk normality test (*W*_(1,1000)_ = 0.999, *p* = 0.953). Relative to this distribution, the fit of the unshuffled normalized theta frequency at max power dataset was more than two standard deviations outside of the mean.

#### Experiment Two—RAT Frequency when Food is Delivered at 6 pm and Animals begin Recording at Various Times

In the first analysis, all datasets were aligned to the time of entry of the animal into the environment, and hence the start of the recording. This disrupted any temporal connection to the timing of food availability. The PSD of the normalized RAT frequency data time series revealed that the greatest energy (within the 12.5 and 50 h window tested) occurred at a period of 50 h with no clear peak in the PSD near 25 h that would indicate circadian rhythmicity. We then smoothed the data with a 5 point moving average before further testing using circular statistics (Moore’s modified Rayleigh test). RAT frequency was represented as vector length at each phase angle. The results indicated no significant periodicity (*R* = 0.57, *p* > 0.1).

In the second analysis, data were aligned to the regular time of feeding (6 pm). The PSD of the normalized RAT frequency data time series revealed that the greatest the energy (within the 12.5 and 50 h window tested) occurred at a period of 28 h. We interpreted this as initial evidence of circadian rhythmicity and then smoothed the data with a 5 point moving average before further testing for periodicity at 25 h with Moore’s modified Rayleigh test, where frequency was represented as vector length at each phase angle. The results indicated significant periodicity (*R* = 1.03, *p* < 0.05) with a mean phase angle of 65 degrees, placing the acrophase at around 4 h after recording start.

**Figure 2:**
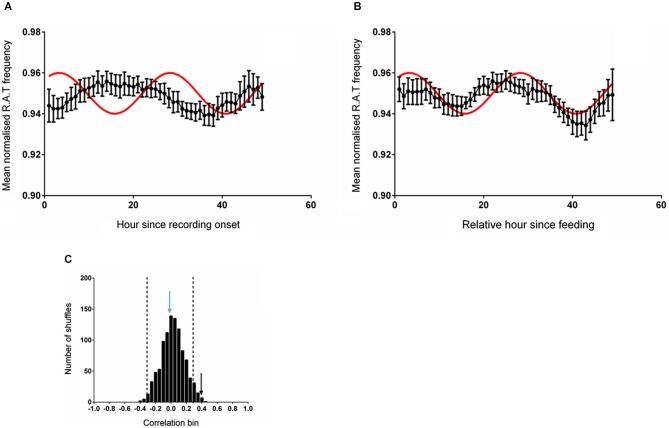
**(A)** The mean (±sem) normalized RAT frequency data obtained when individual recordings are referenced to entry into the recording apparatus, but not the time of feeding, are not correlated with the reference sine shown in red. **(B)** When the individual RAT frequency data are shifted such that they are referenced to the time of feeding (6 pm solar time), the mean data are significantly correlated with the reference (*p* < 0.05). Data are smoothed using a 5-point moving average for these illustrations only. **(C)** illustrates the frequency distribution produced when 1000 shuffled versions of the dataset are generated and correlated against the reference sine. The vertical dashed lines demark two standard deviations from the mean of the distribution. The vertical blue arrow denotes the magnitude of the correlation of the dataset shown in **(A)** and the vertical black arrow denotes the magnitude of the correlation of the dataset shifted relative to a feeding reference in **(B)**.

The mean (unsmoothed) data from the two alignments were then compared to the reference sine wave. The normalized RAT frequency for these two analyses and the corresponding sine fit are illustrated in Figure 2. When data were aligned to entry to the environment, there was no significant correlation between normalized RAT frequency and the sine reference (*r* = 0.110, *p* = 0.450, n.s; Figure 2A). When the data were individually phase-shifted to align with regular food time, the mean time-series was significantly correlated with the reference sine frequency (*r* = 0.307, *p* = 0.025; Figure 2B).

The distribution of the correlations obtained from fitting the reference sine to 1000 randomly shuffled versions of these datasets is illustrated in Figure 2C. The distribution of shuffled correlations is normal with a mean near zero (Shapiro-Wilk normality test frequency: *W*_(1,1000)_ = 0.999, *p* = 0.953). In both cases, the correlation between the reference sine and the actual frequency time-series of RAT were more than two standard deviations outside the mean of the shuffled data, indicating that the relationship was unlikely to have occurred by chance.

#### Experiment Three—RAT Frequency when Food is Available Two Hours after the Start of Recording Irrespective of Start Time

**Figure 3:**
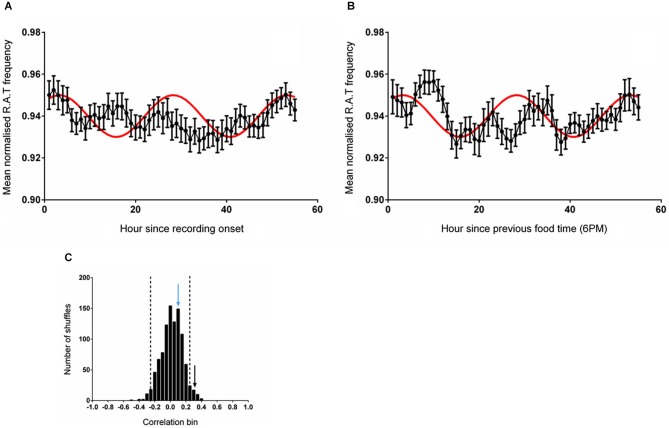
**(A)** The mean (±sem) normalized RAT frequency when food is given at hour 2 of recording and the data are referenced to the entry of the animal into the recording apparatus. **(B)** Illustrates the mean normalized RAT frequency when food is given at hour 2 of recording, but the data are referenced to the expected food time (6 pm). The frequency distribution produced by generating 1000 shuffled versions of the frequency dataset is illustrated in **(C)**. The vertical dashed lines indicate two standard deviations from the mean of the distribution. The vertical black arrow represents the magnitude of the correlation of the data shown in **(A)** against the reference sine, while the vertical blue arrow represents the magnitude of the correlation of the data illustrated in **(B)** against the reference sine (shown in red).

In this experiment, time-series data were either shifted to be aligned to entry into the environment (which also coincided with a food delivery event) or aligned to regular feeding time (6 pm). The mean RAT frequency was compared with the reference wave as described previously. As illustrated in Figure 3A, the frequency of RAT was significantly correlated (*r* = 0.331, *p* = 0.011) with the reference sine when the data were aligned to environment entry (and therefore also to food delivery). When the data were aligned to the previous regular feeding time, however, the relationship was eliminated (Frequency, *r* = 0.108, *p* = 0.415, n.s; Figure 3B). As in the previous experiments, 1000 randomly shuffled versions of the dataset were constructed and fitted to the reference sine. A Shapiro-Wilk normality test confirmed the normality of these fits (Frequency, *W*_(1,1000)_ = 0.999, *p* = 0.624). The correlation coefficient of RAT frequency when the data was aligned with the start of recording (hour 1) were more than two standard deviations outside the mean of the shuffled distribution (Figure 3C).

### Cosinor Analysis

**Figure 4:**
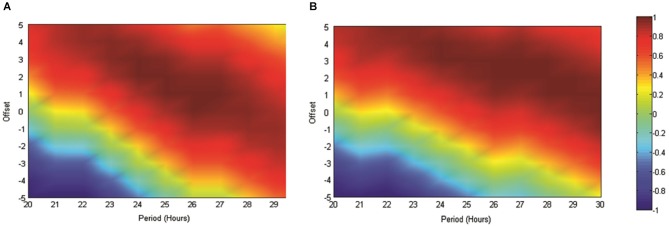
**(A)** The matrix produced by correlating an array of sinusoids with period *x* and offset *y* hours against the artificially generated sine wave (25 h period and 2 h offset) used as a reference throughout this study. As indicated in the scale, “hotter” colors represent increasingly positive correlations, while cooler colors represent increasingly negative correlations. **(B)** Illustrates the correlation matrix produced by correlating an array of sinusoids with period *x* and offset *y* against the mean normalized spontaneously generated hippocampal theta frequency.

**Figure 5:**
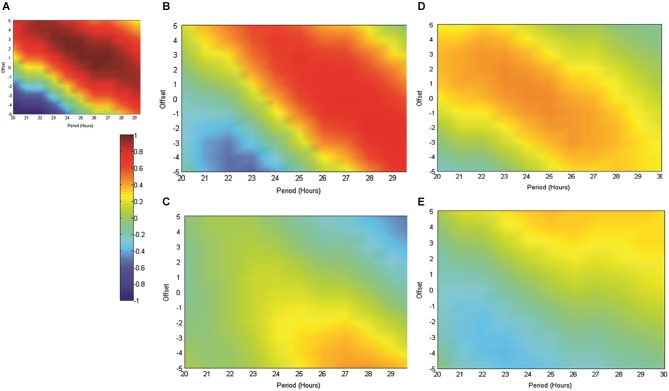
**(A)** The matrix produced by correlating an array of sinusoids with period *x* and offset *y* hours against the artificially generated sine wave used as a reference throughout this study (as in **4A**). As indicated in the scale, “hotter” colors represent increasingly positive correlations, while “cooler” colors represent increasingly negative correlations. **(B)** illustrates the matrix produced when the mean normalized normalized RAT frequency is correlated against sinusoids of varying period and offset and when individual datasets are referenced to the normal feeding time at 6 pm. In **(C)**, data are referenced to the start of the recording, which since it varied across the day, removes any association with the feeding time. **(D,E**) illustrate the matrices produced when mean RAT frequency is correlated against sines of varying period and offset. For these datasets, food was made available on hour 2 of the recording and the data are shown either **(D)** referenced to recording start, or shifted so as to be referenced to the time of day, and by implication normal feeding time at 6 pm **(E)**.

In order to further validate our use of the reference sinusoid in this dataset we conducted cosinor analyses of all RAT data in MATLAB as described in Nelson et al. (1979). For Experiment 2, when food was made available at 6 pm and data were referenced against this feeding time, good fits to the data were obtained with periods of between 20–30 h and varying acrophase. The best fit with a 24 h period placed the acrophase at approximately 4 h into recording, at 25 h the lag was 3 h and at 26 h, 2 h. This result was both consistent with our previous data and also indicated a strong circadian effect. In contrast, when these data were not referenced to feeding time, but rather entry into the environment, the cosinor fits were markedly poorer around the circadian period. In Experiment 3, the best fits to the dataseries obtained with alignment to food delivery in the environment, were obtained with periods of between 23 and 25 h and acrophases of 2.2–0.5 h. Markedly poorer fits were obtained when the data were aligned to previous food delivery time. For purposes of visualization we have shown how these fits vary in Figures 4, 5. Figure 4A illustrates the correlation matrix (hotter colors representing stronger correlations) produced by correlating reference waveforms with period *x* and offset *y* (where *x* varies from 20–30 h and *y* varies from +5 to −5 h, with a positive offset indicating an initial peak occurring after recording start) against the sine comparison used throughout the previous extent of this study. As expected, the highest correlation is obtained where the waveforms match exactly (25 h wavelength, 2 h offset) but note that this procedure also generates a diagonal band of high correlations as period and offset interact indicating the difficulty in tightly specifying a period and acrophase when the dataset consists of relatively few cycles. Figure 4B shows that the correlation matrix produced when fits are made to the free-running theta frequency data obtained in experiment one, and illustrated in Figure 1A, is very similar to this reference, indicating a good fit obtained at the reference 25 h period and 2 h offset. The upward shift of the high-correlation band compared to Figure 4A indicates that a reference with a slightly longer period or offset also provide very good fits to the data.

Figure 5 illustrates the correlations between RAT frequency data obtained in experiment two and three and reference sinusoids of varying period and offset. For reference purposes Figure 5A illustrates the correlation matrix produced by a pure 25 h period, 2 h-offset, sine wave. **5B** illustrates the correlation matrix produced with RAT when food is delivered at 6 pm and data are shifted so that all recordings are time-referenced to 6 pm, irrespective of their actual recording time. It can be seen that there is a good fit to a 25 h period, 2 h offset, reference with a characteristic diagonal band of relatively high correlations. In contrast, if the data are not time-shifted, such that each recording has no systematic relationship to feeding time, correlations with any potential reference are markedly reduced and there is little evidence of the banding produced by correlations against sinusoidal data (Figure 5A). Figure 5D illustrates the correlation matrix produced to the RAT frequency data when food is always presented 2 h into recording (experiment three) and the data-series are not shifted. There is a band of higher correlation that runs through the reference period/offset points and banding indicative of sinusoidal data. In contrast, when the individual time-series of this set of rats RAT frequency data are shifted such that 6 pm, the previously “normal” feeding time, is the reference point, there was poor correlation between the mean dataset and reference sinusoids any period and offset tested (Figure 5E).

## Discussion

The present experiments provide clear evidence that the frequency of hippocampal theta rhythm in free moving animals is modulated on a circadian timescale in much the same manner as has been reported for the activity of hippocampal single units (Munn and Bilkey, 2012). Furthermore, changes in free-running theta frequency and single unit firing rate were strongly related over these periods indicating that the EEG activity covaries with firing rate. For this reason we performed the majority of our analyses using the same reference as we used in our previous study (Munn and Bilkey, 2012); a sine wave with 25 h frequency and 2 h offset. This allowed us to test a tightly-constrained *a priori* hypothesis. The fact that we find periodicity in our data with these parameters does not, however, mean that good fits could not be obtained for other periods and acrophases that are near this value. In fact, the cosinor analysis demonstrates this. We cannot, therefore, rule out the possibility that further analysis, with greater *n* or experiment duration, might reveal that the actual period of best fit, and/or offset, is one or two hours different from that which we have described.

This aside, the data are clearly circadian-modulated by standard measures and so a key question in deciphering the function of the observed oscillations is to determine what stimuli entrain these rhythms. In the aforementioned single unit study, time-of solar-day and light were eliminated as possible entraining stimuli. Food and environmental novelty were possible alternative entraining stimuli but in the previous study they were inextricably linked; animals must cover the majority of the environment in order to capture place cell firing and this is typically (and best) done by motivating a food-deprived animal with scattered food. The finding that theta frequency and single unit firing rate were correlated allowed us to use a novel artificial theta elicitation paradigm that explicitly requires animals to remain motionless to clearly disambiguate food from environmental novelty. This paradigm also enabled the use of very long recording sessions spanning more than two solar days. In contrast, recordings of naturally elicited theta over periods of longer than one day are problematic due to the tendency for animals not to move much after many hours of exposure to the same environment.

While RAT is not “natural” theta it shares many of the same mechanisms and features. It has previously been shown that the reticular formation is a key region in the ascending spontaneous theta generation pathway via the medial supramammillary nucleus (Bland, 1986; Kirk and McNaughton, 1991) and the medial septum (Vertes, 1982). Activation of the medial supramammillary nucleus via the reticular formation (and posterior hypothalamus) is thought to provide a frequency code for theta to the medial septum and from there to hippocampus (Kirk, 1998). Furthermore, disconnection of the reticular formation from the medial septum reduces normal theta-burst activity in the hippocampus and disorganizes the bursts that do occur (Vinogradova, 1995). Conversely, both natural (sensory) stimulation and direct electrical stimulation of the reticular formation increase both the frequency of theta-bursting and number of neurons that display bursting in the diagonal band of the medial septum. (Vinogradova et al., 1998). Stimulation of the reticular formation thereby provides a novel means of controlling the mechanisms involved in the generation of endogenous theta.

Using the RAT paradigm, we show a clear double dissociation between the availability of food and entry into a novel environment as entraining stimuli for the circadian modulation of theta activity. By separating feeding time from experiment start time in one experiment and making feeding time coincident with start time in the other, the effects of entry into the recording environment and the availability of food were able to be separately examined and food availability was shown to be the entraining factor. Furthermore, our data are consistent with the findings of our previous study in showing that these circadian oscillations were not entrained to time of day, ruling out the possibility that the observed modulation could be due to changes in behavioral state associated with solar time. The finding that food is the entraining stimulus for theta, and by inference, hippocampal cell activity, is consistent with previous data suggesting that the hippocampus may respond to biofeedback cues associated with food intake (Carlini et al., 2004) and may monitor energy state through these cues (Carlini et al., 2002, 2004). Biofeedback hormones play a complex role in receptor expression for other hormones in hippocampus, but only when paired with a learning task (Paulus et al., 2005), suggesting a specific role for these food-related hormones in learning and memory. There is other evidence that the hippocampus is part of a circadian oscillator that is entrained by food; c-Fos expression in hippocampus is modulated by restricted feeding (Tracy et al., 2001; Poulin and Timofeeva, 2008), and Wakamatsu et al. (2001) demonstrated that clock genes in the hippocampus are phase-shifted in response to restricted feeding.

Since the data indicate that the activity of the hippocampus can oscillate on a circadian timescale, and that this oscillation is able to be entrained by the availability of food, it is of interest to examine the similarities between this phenomenon and putative mechanisms in the brain controlling food biofeedback. There has been considerable recent theoretical and experimental work investigating the existence and function of the so-called “food entrainable oscillator” (FEO; Angeles-Castellanos et al., 2005, 2007; Davidson et al., 2001b, 2003; Martinez et al., 2001; Carneiro and Araujo, 2009; Mistlberger, 2011). This oscillator has a circadian period but exists separately from the well-described light-entrained oscillator in the suprachiasmatic nucleus. When the SCN is ablated, circadian rhythmicity in behavior persists (Davidson and Stephan, 1999; Landry et al., 2006). Food-entrained circadian behavior also persists in animals that have some disruptions in the “clock gene” system recruited by the SCN (Pitts et al., 2003). There has been considerable conjecture as to the precise location of the FEO; there have been suggestions that it is driven by oscillators in the periphery (Escobar et al., 2009), but lesion studies have so far failed to show that the FEO is instantiated in any single structure (Comperatore and Stephan, 1990; Davidson et al., 2001a,b; Davidson, 2009; Moriya et al., 2009). The hippocampus has, however, been implicated in this process. For example, lesioned animals display difficulty representing time over short time scales (Meck et al., 2013). On longer timescales, Davidson et al. (2010) showed that while intact animals were successfully able to solve a discrimination problem based on feeding time, animals that had received lesions of the hippocampus, were not. The results of this latter study, however, are not definitive evidence that the hippocampus is necessary for the representation of circadian information; the results may be explained by the inability of lesioned animals to form any kind of temporal trace memory. Molecular studies provide evidence that the hippocampus is at least equipped with the necessary cellular mechanisms to represent circadian time; the clock genes that are central to the rhythmicity of the suprachiasmatic nucleus are represented in hippocampus, oscillate on a circadian frequency, and shift relative to the anticipation of food (Angeles-Castellanos et al., 2007; Wang et al., 2009). It is unclear, however, exactly what effect these genes may have on cognition and behavior; although Bowden et al. (2011) demonstrated that the induction of hippocampal plasticity was influenced by time of day, raising the possibility that circadian rhythmicity in the hippocampus is linked to memory encoding and/or retrieval.

A food entrained oscillation in the activity of the hippocampus may underlie a system in which the availability of food triggers the initiation of a state (high firing rate) in the hippocampus that reoccurs approximately 24 h later. The high firing rate may facilitate synaptic plasticity at these times or may simply reflect a state in which the processing capabilities of the hippocampus are greater. Alternatively, a higher activity state may function to facilitate the return of an animal to a location where food might be available on a circadian cycle. A mechanism via which information about the spatial locations of food goals is available with salience modulated on a circadian period is ethologically attractive. The foraging behavior of many organisms is organized on a circadian timescale, in some cases to reflect changes in food availability during the day (Bachman, 1984; Croll et al., 1992). While modulating food seems to be a sufficient stimulus for entraining hippocampal theta, it is important to note that the animals in these experiments are strongly food-deprived. Food is therefore a salient stimulus. In fact, apart from the environmental novelty caused by transportation from their home cage to the recording apparatus, food is perhaps the only salient stimulus that occurs during recording. Therefore, while the activity of the hippocampus is entrained by food under these circumstances, it is possible that any sufficiently salient stimulus could have the capacity to entrain the circadian modulation. This could reflect a more general memorial function for the circadian oscillations in theta frequency, perhaps reflecting increased epochs of encoding and retrieval at the time of stimulus availability and then again when the animal is temporally proximal to the time of day that the salient stimulus originally occurred. Such a mechanism would be in agreement with models predicting, and data demonstrating, that theta may “gate” the hippocampus into encoding and retrieval epochs (Huerta and Lisman, 1996; Hasselmo et al., 2002; Hyman et al., 2003). Higher theta frequency in concert with higher single unit firing rates as we have previously observed (Munn and Bilkey, 2012) could thereby increase the temporal resolution of encoding and retrieval, offering more encoding and retrieval chances per unit time.

The results of the present experiments demonstrate that the availability of food has the capacity to entrain the circadian modulation of hippocampal activity, and it is therefore possible that the hippocampus is a central structure in the putative FEO. It is unclear, however, which specific biofeedback mechanism (if any) might drive the entrainment event. One candidate hormone is leptin, a hormone that is released by adipocytes in response to fat ingestion. The basal expression of leptin is diurnal and can be entrained by food (Martinez et al., 2001), hippocampal neurons are responsive to leptin (Shanley et al., 2001), and leptin has also been shown to affect LTP in the hippocampus *in vitro* (Oomura et al., 2006; Moult et al., 2009). Furthermore, Ob/Ob mice, which are leptin resistant, tend to show abnormal circadian modulation of activity (Sans-Fuentes et al., 2010). The ingestion of food may therefore entrain the FEO-like signal observed in the hippocampus through the release of leptin. This may serve to regulate internal state in order to drive an animal to “remember” when (on a circadian scale) and where food was available, in order to enable successful foraging.

The finding that hippocampal circadian activity is entrained by food-reward events may also be relevant to recent work focusing on a role for the hippocampus in anxiety (McNaughton and Gray, 2000; Bannerman et al., 2004; Engin and Treit, 2007). The non-availability of food is linked to anxiety, and anxiety disorders are often comorbid with disorders of eating (Swinbourne et al., 2012). Furthermore, leptin has been proposed to have anxiolytic functions (Asakawa et al., 2003; Liu et al., 2010). This raises the interesting possibility that the non-availability of food promotes anxiety which then adaptively increase foraging (Rosenblum and Paully, 1984). Since attaining food triggers leptin release, this event could theoretically reset the FEO, both decreasing anxiety and hippocampal activity. A leptin-triggered FEO reset would reduce energy-consuming foraging behavior, mediated either through changes in anxiety or through hippocampal control of the behavior itself, for a period during which such behavior would no longer be adaptive. Such an account, while speculative, might move somewhat towards an integration of the anxiety (Gray and Mcnaughton, 2000) and spatial (O’Keefe and Dostrovsky, 1971) accounts of hippocampal function, and provides avenues for the integration of anxiety disorders, and disorders of eating such as anorexia and obesity. If disorders of eating and anxiety are fundamentally linked at the level of the hippocampus, potentially novel treatments for both types of disorder might be found.

In summary, we present evidence that the activity of the hippocampus, a structure central to episodic memory, is modulated over timescales that closely map onto a complete circadian cycle. Furthermore, we show that this oscillation in activity appears to be entrained by the availability of food rather than time of day or exposure to the recording environment. These findings suggest that the hippocampus could encode temporal information over much longer timescales than previously shown and suggest that a FEO could modulate both foraging behavior and memory performance over circadian periods.

## Conflict of Interest Statement

The authors declare that the research was conducted in the absence of any commercial or financial relationships that could be construed as a potential conflict of interest.

## References

[B1] Aggleton J. P. Brown M. W. (1999). Episodic memory, amnesia and the hippocampal-anterior thalamic axis. Behav. Brain Sci. 22, 425–444. 10.1017/s0140525x99002034 11301518

[B2] Angeles-Castellanos M. Mendoza J. Díaz-Muñoz M. Escobar C. (2005). Food entrainment modifies the c-Fos expression pattern in brain stem nuclei of rats. Am. J. Physiol. Regul. Integr. Comp. Physiol. 288, R678–R684. 10.1152/ajpregu.00590.2004 15550615

[B3] Angeles-Castellanos M. Mendoza J. Escobar C. (2007). Restricted feeding schedules phase shift daily rhythms of c-Fos and protein Per1 immunoreactivity in corticolimbic regions in rats. Neuroscience 144, 344–355. 10.1016/j.neuroscience.2006.08.064 17045749

[B4] Asakawa A. Inui A. Inui T. Katsuura G. Fujino M. A. Kasuga M. (2003). Leptin treatment ameliorates anxiety in ob/ob obese mice. J. Diabetes Complications 17, 105–107. 10.1016/s1056-8727(02)00185-x 12614977

[B5] Bachman R. A. (1984). Foraging behavior of free-ranging wild and hatchery brown trout in a stream. Trans. Am. Fish. Soc. 113, 1–32 10.1577/1548-8659(1984)113<1:fbofwa>2.0.co;2

[B6] Bannerman D. M. Rawlins J. N. P. Mchugh S. B. Deacon R. M. J. Yee B. K. Bast T.. (2004). Regional dissociations within the hippocampus–memory and anxiety. Neurosci. Biobehav. Rev. 28, 273–283. 10.1016/j.neubiorev.2004.03.004 15225971

[B7] Barry J. Muller R. (2011). Updating the hippocampal representation of space: place cell firing fields are controlled by a novel spatial stimulus. Hippocampus 21, 481–494. 10.1002/hipo.20764 20087890

[B8] Biebach H. Falk H. Krebs J. R. (1991). The effect of constant light and phase shifts on a learned time-place association in garden warblers (Sylvia borin): hourglass or circadian clock? J. Biol. Rhythms 6, 353–365. 10.1177/074873049100600406 1773101

[B9] Bilkey D. K. Muir G. M. (1999). A low cost, high precision subminiature microdrive for extracellular unit recording in behaving animals. J. Neurosci. Methods 92, 87–90. 10.1016/s0165-0270(99)00102-8 10595706

[B10] Bland B. H. (1986). The physiology and pharmacology of hippocampal formation theta rhythms. Prog. Neurobiol. 26, 1–54. 10.1016/0301-0082(86)90019-5 2870537

[B11] Bostock E. Muller R. U. Kubie J. L. (1991). Experience-dependent modifications of hippocampal place cell firing. Hippocampus 1, 193–205. 10.1002/hipo.450010207 1669293

[B12] Bowden J. B. Abraham W. C. Harris K. M. (2011). Differential effects of strain, circadian cycle and stimulation pattern on LTP and concurrent LTD in the dentate gyrus of freely moving rats. Hippocampus 22, 1363–1370. 10.1002/hipo.20972 21853503 PMC3292688

[B13] Buzsàki G. Eidelberg E. (1983). Phase-Relations of hippocampal projection cells and interneurons to theta activity in the anesthetized rat. Brain Res. 266, 334–339. 10.1016/0006-8993(83)90665-0 6191827

[B14] Carlini V. P. Monzón M. E. Varas M. M. Cragnolini A. B. Schiöth H. B. Scimonelli T. N.. (2002). Ghrelin increases anxiety-like behavior and memory retention in rats. Biochem. Biophys. Res. Commun. 299, 739–743. 10.1016/s0006-291x(02)02740-7 12470640

[B15] Carlini V. P. Varas M. M. Cragnolini A. B. Schiöth H. B. Scimonelli T. N. De Barioglio S. R. (2004). Differential role of the hippocampus, amygdala and dorsal raphe nucleus in regulating feeding, memory and anxiety-like behavioral responses to ghrelin. Biochem. Biophys. Res. Commun. 313, 635–641. 10.1016/j.bbrc.2003.11.150 14697239

[B16] Carneiro B. T. Araujo J. F. (2009). The food-entrainable oscillator: a network of interconnected brain structures entrained by humoral signals? Chronobiol. Int. 26, 1273–1289. 10.3109/07420520903404480 19916831

[B17] Comperatore C. A. Stephan F. K. (1990). Effects of vagotomy on entrainment of activity rhythms to food access. Physiol. Behav. 47, 671–678. 10.1016/0031-9384(90)90076-g 2385637

[B18] Croll D. A. Gaston A. J. Burger A. E. Konnoff D. (1992). Foraging behavior and physiological adaptation for diving in thick-billed murres. Ecology 73, 344–356 10.2307/1938746

[B19] Davidson A. J. (2009). Lesion studies targeting food-anticipatory activity. Eur. J. Neurosci. 30, 1658–1664. 10.1111/j.1460-9568.2009.06961.x 19863659

[B20] Davidson A. J. Aragona B. J. Houpt T. A. Stephan F. K. (2001a). Persistence of meal-entrained circadian rhythms following area postrema lesions in the rat. Physiol. Behav. 74, 349–354. 10.1016/s0031-9384(01)00567-4 11714499

[B21] Davidson A. J. Aragona B. J. Werner R. M. Schroeder E. Smith J. C. Stephan F. K. (2001b). Food-anticipatory activity persists after olfactory bulb ablation in the rat. Physiol. Behav. 72, 231–235. 10.1016/s0031-9384(00)00417-0 11240001

[B22] Davidson T. L. Jarrard L. E. (1993). A role for hippocampus in the utilization of hunger signals. Behav. Neural Biol. 59, 167–171. 10.1016/0163-1047(93)90925-8 8476385

[B23] Davidson T. L. Kanoski S. E. Chan K. Clegg D. J. Benoit S. C. Jarrard L. E. (2010). Hippocampal lesions impair retention of discriminative responding based on energy state cues. Behav. Neurosci. 124, 97–105. 10.1037/a0018402 20141284 PMC2850045

[B24] Davidson A. J. Poole A. S. Yamazaki S. Menaker M. (2003). Is the food-entrainable circadian oscillator in the digestive system? Genes Brain Behav. 2, 32–39. 10.1034/j.1601-183x.2003.00005.x 12882317

[B25] Davidson A. J. Stephan F. K. (1999). Feeding-entrained circadian rhythms in hypophysectomized rats with suprachiasmatic nucleus lesions. Am. J. Physiol. 277, R1376–R1384. 10564210 10.1152/ajpregu.1999.277.5.R1376

[B26] Edmonds S. C. Adler N. T. (1977). Food and light as entrainers of circadian running activity in rat. Physiol. Behav. 18, 915–919. 10.1016/0031-9384(77)90201-3 905400

[B27] Eichenbaum H. (2014). Time cells in the hippocampus: a new dimension for mapping memories. Nat. Rev. Neurosci. 15, 732–744. 10.1038/nrn3827 25269553 PMC4348090

[B28] Eichenbaum H. Fortin N. (2003). Episodic memory and the hippocampus: it’s about time. Curr. Dir. Psychol. Sci. 12, 53–57 10.1111/1467-8721.01225

[B29] Engin E. Treit D. (2007). The role of hippocampus in anxiety: intracerebral infusion studies. Behav. Pharmacol. 18, 365–374. 10.1097/fbp.0b013e3282de7929 17762507

[B30] Escobar C. Cailotto C. Angeles-Castellanos M. Delgado R. S. Buijs R. M. (2009). Peripheral oscillators: the driving force for food-anticipatory activity. Eur. J. Neurosci. 30, 1665–1675. 10.1111/j.1460-9568.2009.06972.x 19878276

[B31] Fortin N. J. Agster K. L. Eichenbaum H. B. (2002). Critical role of the hippocampus in memory for sequences of events. Nat. Neurosci. 5, 458–462. 10.1038/nn834 11976705 PMC4053170

[B32] Foster D. J. Wilson M. A. (2007). Hippocampal theta sequences. Hippocampus 17, 1093–1099. 10.1002/hipo.20345 17663452

[B33] Fox S. E. Wolfson S. Ranck J. B. (1986). Hippocampal theta-rhythm and the firing of neruons in walking and urethane anesthetized rats. Exp. Brain Res. 62, 495–508. 10.1007/bf00236028 3720881

[B34] Gray J. A. Mcnaughton N. (2000). The Neuropsychology of Anxiety. 2nd Edn. Oxford: Oxford University Press.

[B35] Hasselmo M. E. (2005). What is the function of hippocampal theta Rhythm? Linking behavioral data to phasic properties of field potential and unit recording data. Hippocampus 15, 936–949. 10.1002/hipo.20116 16158423

[B36] Hasselmo M. E. Bodelón C. Wyble B. P. (2002). A proposed function for hippocampal theta rhythm: separate phases of encoding and retrieval enhance reversal of prior learning. Neural Comput. 14, 793–817. 10.1162/089976602317318965 11936962

[B37] Hoge J. Kesner R. P. (2007). Role of CA3 and CA1 subregions of the dorsal hippocampus on temporal processing of objects. Neurobiol. Learn. Mem. 88, 225–231. 10.1016/j.nlm.2007.04.013 17560815 PMC2095779

[B38] Hok V. Lenck-Santini P. P. Roux S. Save E. Muller R. U. Poucet B. (2007). Goal-related activity in hippocampal place cells. J. Neurosci. 27, 472–482. 10.1523/jneurosci.2864-06.2007 17234580 PMC6672791

[B39] Honma K.-I. Hiroshige T. (1978). Internal synchronization among several circadian rhythms in rats under constant light. Am. J. Physiol. 235, R243–R249. 727286 10.1152/ajpregu.1978.235.5.R243

[B40] Huerta P. T. Lisman J. E. (1996). Low-frequency stimulation at the troughs of theta-oscillation induces long-term depression of previously potentiated CA1 synapses. J. Neurophysiol. 75, 877–884. 8714660 10.1152/jn.1996.75.2.877

[B41] Hyman J. M. Wyble B. P. Goyal V. Rossi C. A. Hasselmo M. E. (2003). Stimulation in hippocampal region CA1 in behaving rats yields long-term potentiation when delivered to the peak of theta and long-term depression when delivered to the trough. J. Neurosci. 23, 11725–11731. 14684874 10.1523/JNEUROSCI.23-37-11725.2003 PMC6740943

[B42] Jackson T. P. (2001). Factors influencing food collection behaviour of Brants’ whistling rat (Parotomys brantsii): a central place forager. J. Zool. 255, 15–23 10.1017/s0952836901001078

[B43] Kesner R. P. Hunsaker M. R. (2010). The temporal attributes of episodic memory. Behav. Brain Res. 215, 229–309. 10.1016/j.bbr.2009.12.029 20036694

[B44] Kirk I. J. (1998). Frequency modulation of hippocampal theta by the supramammillary nucleus and other hypothalamo-hippocampal interactions: mechanisms and functional implications. Neurosci. Biobehav. Rev. 22, 291–302. 10.1016/s0149-7634(97)00015-8 9579319

[B45] Kirk I. J. McNaughton N. (1991). Supramammillary cell firing and hippocampal rhythmical slow activity. Neuroreport 2, 723–725. 10.1097/00001756-199111000-00023 1810464

[B46] Landry G. J. Simon M. M. Webb I. C. Mistlberger R. E. (2006). Persistence of a behavioral food-anticipatory circadian rhythm following dorsomedial hypothalamic ablation in rats. Am. J. Physiol. Regul. Integr. Comp. Physiol. 290, R1527–R1534. 10.1152/ajpregu.00874.2005 16424080

[B47] Liu J. Garza J. C. Bronner J. Kim C. S. Zhang W. Lu X.-Y. (2010). Acute administration of leptin produces anxiolytic-like effects: a comparison with fluoxetine. Psychopharmacology (Berl) 207, 535–545. 10.1007/s00213-009-1684-3 19823809 PMC4057895

[B48] MacDonald C. J. Lepage K. Q. Eden U. T. Eichenbaum H. (2011). Hippocampal “time cells” bridge the gap in memory for discontiguous events. Neuron 71, 737–749. 10.1016/j.neuron.2011.07.012 21867888 PMC3163062

[B49] Martinez T. M. Parra-Gamez L. G. Aguilar R. R. Escobar C. B. (2001). Leptin rhythmicity is entrained to feeding schedules in rats. Soc. Neurosci. Abstr. 27, 486.

[B50] McNaughton N. Gray J. A. (2000). Anxiolytic action on the behavioural inhibition system implies multiple types of arousal contribute to anxiety. J. Affect. Disord. 61, 161–176. 10.1016/s0165-0327(00)00344-x 11163419

[B51] McNaughton N. Richardson J. Gore C. (1986). Reticular elicitation of hippocampal slow waves: common effects of some anxiolytic drugs. Neuroscience 19, 899–903. 10.1016/0306-4522(86)90306-4 2879256

[B52] McNaughton N. Sedgwick E. M. (1978). Reticular stimulation and hippocampal theta rhythm in rats: effects of drugs. Neuroscience 3, 629–632. 10.1016/0306-4522(78)90004-0 724111

[B53] Meck W. H. Church R. M. Olton D. S. (1984). Hippocampus, time and memory. Behav. Neurosci. 98, 3–22. 10.1037//0735-7044.98.1.3 6696797

[B54] Meck W. H. Church R. M. Olton D. S. (2013). Hippocampus, time and memory. Behav. Neurosci. 127, 655–668. 10.1037/a0034188 24128355

[B55] Mendoza J. Albrecht U. Challet E. (2010). Behavioural food anticipation in clock genes deficient mice: confirming old phenotypes, describing new phenotypes. Genes Brain Behav. 9, 467–477. 10.1111/j.1601-183x.2010.00576.x 20180860

[B56] Meyers-Manor J. E. Overmier J. B. Hatfield D. W. Croswell J. (2014). Not so bird-brained: Pigeons show what-where-when memory both as time of day and how long ago. J. Exp. Psychol. Anim. Learn. Cogn. 40, 225–240. 10.1037/xan0000016 24377432

[B57] Mistlberger R. E. (2006). Circadian rhythms: perturbing a food-entrained clock. Curr. Biol. 16, R968–R969. 10.1016/j.cub.2006.10.020 17113381

[B58] Mistlberger R. E. (2011). Neurobiology of food anticipatory circadian rhythms. Physiol. Behav. 104, 535–545. 10.1016/j.physbeh.2011.04.015 21527266

[B59] Moriya T. Aida R. Kudo T. Akiyama M. Doi M. Hayasaka N.. (2009). The dorsomedial hypothalamic nucleus is not necessary for food-anticipatory circadian rhythms of behavior, temperature or clock gene expression in mice. Eur. J. Neurosci. 29, 1447–1460. 10.1111/j.1460-9568.2009.06697.x 19519629

[B60] Moult P. R. Milojkovic B. Harvey J. (2009). Leptin reverses long-term potentiation at hippocampal CA1 synapses. J. Neurochem. 108, 685–696. 10.1111/j.1471-4159.2008.05810.x 19054283 PMC2638023

[B61] Mumby D. G. Gaskin S. Glenn M. J. Schramek T. E. Lehmann H. (2002). Hippocampal damage and exploratory preferences in rats: memory for objects, places and contexts. Learn. Mem. 9, 49–57. 10.1101/lm.41302 11992015 PMC155935

[B62] Munn R. G. Bilkey D. K. (2012). The firing rate of hippocampal CA1 place cells is modulated with a circadian period. Hippocampus 22, 1325–1337. 10.1002/hipo.20969 21830249

[B63] Munn R. G. K. McNaughton N. (2008). Effects of fluoxetine on hippocampal rhythmic slow activity and behavioural inhibition. Behav. Pharmacol. 19, 257–264. 10.1097/fbp.0b013e3282ff1300 18469543

[B64] Nelson W. Tong Y. L. Lee J. K. Halberg F. (1979). Methods for cosinor-rhythmometry. Chronobiologia 6, 305–323. 548245

[B65] O’Keefe J. Conway D. H. (1978). Hippocampal place units in freely moving rat: why they fire where they fire. Exp. Brain Res. 31, 573–590. 10.1007/bf00239813 658182

[B66] O’Keefe J. Dostrovsky J. (1971). The hippocampus as a spatial map. Preliminary evidence from unit activity in the freely-moving rat. Brain Res. 34, 171–175. 10.1016/0006-8993(71)90358-1 5124915

[B67] O’Keefe J. Nadel L. (1978). The Hippocampus as a Cognitive Map. Oxford: Clarendon Press.

[B68] O’Keefe J. Recce M. L. (1993). Phase relationship between hippocampal place units and the EEG theta rhythm. Hippocampus 3, 317–330. 10.1002/hipo.450030307 8353611

[B69] Oomura Y. Hori N. Shiraishi T. Fukunaga K. Takeda H. Tsuji M.. (2006). Leptin facilitates learning and memory performance and enhances hippocampal CA1 long-term potentiation and CaMK II phosphorylation in rats. Peptides 27, 2738–2749. 10.1016/j.peptides.2006.07.001 16914228

[B70] Paulus K. Schulz C. Lehnert H. (2005). Central nervous effects of leptin and insulin on hippocampal leptin and insulin receptor expression following a learning task in Wistar rats. Neuropsychobiology 51, 100–106. 10.1159/000084167 15741751

[B71] Paxinos G. Watson C. (1998). Rat Brain in Stereotaxic Coordinates. 4th Edn. San Diego: Academic Press Inc.

[B72] Phillips D. L. Rautenberg W. Rashotte M. E. Stephan F. K. (1993). Evidence for a separate food-entrainable circadian oscillator in the pigeon. Physiol. Behav. 53, 1105–1113. 10.1016/0031-9384(93)90366-n 8346294

[B73] Pitts S. M. Silver R. Sun Z. S. (2003). Circadian gene expression in the liver and forebrain under restricted feeding. Soc. Neurosci. Abstr. Viewer and Itinerary Planner Abstract No. 284.219.

[B74] Poulin A. M. Timofeeva E. (2008). The dynamics of neuronal activation during food anticipation and feeding in the brain of food-entrained rats. Brain Res. 1227, 128–141. 10.1016/j.brainres.2008.06.039 18602903

[B75] Reebs S. G. (1996). Time-place learning in golden shiners (Pisces: Cyprinidae). Behav. Processes 36, 253–262. 10.1016/0376-6357(96)88023-5 24896874

[B76] Rosenblum L. A. Paully G. S. (1984). The effects of varying environmental demands on maternal and infant behavior. Child Dev. 55, 305–314. 10.2307/1129854 6705632

[B77] Sans-Fuentes M. A. Díez-Noguera A. Cambras T. (2010). Light responses of the circadian system in leptin deficient mice. Physiol. Behav. 99, 487–494. 10.1016/j.physbeh.2009.12.023 20060009

[B78] Shanley L. J. Irving A. J. Harvey J. (2001). Leptin enhances NMDA receptor function and modulates hippocampal synaptic plasticity. J. Neurosci. 21:RC186. 11734601 10.1523/JNEUROSCI.21-24-j0001.2001 PMC6763052

[B79] Silver R. Balsam P. (2010). Oscillators entrained by food and the emergence of anticipatory timing behaviors. Sleep Biol. Rhythms 8, 120–136. 10.1111/j.1479-8425.2010.00438.x 21544255 PMC3085253

[B80] Skaggs W. E. McNaughton B. L. Wilson M. A. Barnes C. A. (1996). Theta phase precession in hippocampal neuronal populations and the compression of temporal sequences. Hippocampus 6, 149–172. 10.1002/(sici)1098-1063(1996)6:2<149::aid-hipo6>3.0.co;2-k 8797016

[B81] Stephan F. K. (2002). The “other” circadian system: food as a zeitgeber. J. Biol. Rhythms 17, 284–292. 10.1177/074873002129002591 12164245

[B82] Storch K. F. Weitz C. J. (2009). Daily rhythms of food-anticipatory behavioral activity do not require the known circadian clock. Proc. Natl. Acad. Sci. U S A 106, 6808–6813. 10.1073/pnas.0902063106 19366674 PMC2666092

[B83] Swinbourne J. Hunt C. Abbott M. Russell J. St Clare T. Touyz S. (2012). The comorbidity between eating disorders and anxiety disorders: prevalence in an eating disorder sample and anxiety disorder sample. Aust. N. Z. J. Psychiatry 46, 118–131. 10.1177/0004867411432071 22311528

[B84] Tracy A. L. Jarrard L. E. Davidson T. L. (2001). The hippocampus and motivation revisited: appetite and activity. Behav. Brain Res. 127, 13–23. 10.1016/s0166-4328(01)00364-3 11718882

[B85] Vertes R. P. (1982). Brain stem generation of the hippocampal EEG. Prog. Neurobiol. 19, 159–186. 10.1016/0301-0082(82)90005-3 6131484

[B86] Verwey M. Amir S. (2009). Food-entrainable circadian oscillators in the brain. Eur. J. Neurosci. 30, 1650–1657. 10.1111/j.1460-9568.2009.06960.x 19863660

[B87] Vinogradova O. S. (1995). Expression, control and probable functional-significance of the neuronal theta-rhythm. Prog. Neurobiol. 45, 523–583. 10.1016/0301-0082(94)00051-i 7624485

[B88] Vinogradova O. S. Kitchigina V. F. Zenchenko C. I. (1998). Pacemaker neurons of the forebrain medical septal area and theta rhythm of the hippocampus. Membr. Cell Biol. 11, 715–725. 9718568

[B89] Wakamatsu H. Yoshinobu Y. Aida R. Moriya T. Akiyama M. Shibata S. (2001). Restricted-feeding-induced anticipatory activity rhythm is associated with a phase-shift of the expression of mPer1 and mPer2 mRNA in the cerebral cortex and hippocampus but not in the suprachiasmatic nucleus of mice. Eur. J. Neurosci. 13, 1190–1196. 10.1046/j.0953-816x.2001.01483.x 11285016

[B90] Wang L. M. C. Dragich J. M. Kudo T. Odom I. H. Welsh D. K. O’Dell T. J.. (2009). Expression of the circadian clock gene Period2 in the hippocampus: possible implications for synaptic plasticity and learned behaviour. ASN Neuro 1:e00012. 10.1042/AN20090020 19570032 PMC2695588

[B91] Wills T. J. Lever C. Cacucci F. Burgess N. O’Keefe J. (2005). Attractor dynamics in the hippocampal representation of the local environment. Science 308, 873–876. 10.1126/science.1108905 15879220 PMC2680068

